# African American English speaking 2nd graders, verbal–*s*, and educational achievement: Event related potential and math study findings

**DOI:** 10.1371/journal.pone.0273926

**Published:** 2022-10-20

**Authors:** J. Michael Terry, Erik R. Thomas, Sandra C. Jackson, Masako Hirotani

**Affiliations:** 1 Department of Linguistics, University of North Carolina at Chapel Hill, Chapel Hill, North Carolina, United States of America; 2 Department of English, North Carolina State University, Raleigh, North Carolina, United States of America; 3 Department of Communication Sciences and Disorders, North Carolina Central University, Durham, North Carolina, United States of America; 4 School of Linguistics and Language Studies, Carleton University, Ottawa, Canada; Pacific Lutheran University, UNITED STATES

## Abstract

A number of influential linguistic analyses hold that African American English (AAE) has no verbal–*s*, the–*s* that, for example, turns *drink* into *drinks* in more mainstream English varieties.On such accounts, sentences like *Mary drinks coffee* are ungrammatical in AAE. Previous behavioral studies suggest that in addition to being ungrammatical, AAE speaking children find these sentences cognitively demanding, and that their presence in mathematical reasoning tests can depress scores. Until now, however, no online sentence processing study nor investigation of neurophysiological markers has been done to support these findings. Aimed at addressing this gap in the literature, the auditory ERP experiment described herein revealed two different processes associated with AAE speaking 2nd graders listening to this type of sentence: a morphosyntactic structure building problem, reflected in a bilateral early anterior-central negativity; and an increase in working memory load, indicated by a bilateral late long-lasting anterior-central negativity. Study participants also took an orally administered test of math word problems. Consistent with previous findings, results showed they answered fewer questions correctly when those questions contained verbal–*s* than when they did not.

## Introduction

American English is not a unitary language variety. Far from being homogenous, it is instead spoken as numerous dialects associated with different regions and income levels, as well as racial and ethnic groups throughout the United States [1]. Recognizing both this linguistic diversity and the social and economic stratification that help create and maintain it, language researchers commonly distinguish between so-called non-standard or non-mainstream dialects, on the one hand, and standard or mainstream dialects, on the other. Non-mainstream dialects are those that like Appalachian English, Latino English, and African American English (AAE), to name only a few, lack widespread institutional support. Mainstream dialects, in contrast, are those that gain position and power from it. A case in point: Labov and Baker (2015) [2] define Standard Classroom English (SCE), the language of instruction in the vast majority of US schools, as “a dialect without any marked regional, local, social class, or ethnic features that may be stigmatized by classroom teachers”.

Over fifty years of linguistic research has established that, despite their being stigmatized by teachers and other language gatekeepers, non-mainstream dialects are not defective versions of their mainstream counterparts. They are not, as too many still believe, illogical error-ridden versions of SCE or any other more mainstream language variety. Rather, they are, in the linguist’s sense of the term, “rule governed systems” in their own rights. Like all dialects, non-mainstream dialects are organized according to internally consistent rule sets–grammars–that are different from, but no less complex or expressive than those of more prestigious language varieties. With respect to AAE, the most studied and, in educational settings, perhaps most stigmatized of all non-mainstream American English dialects, there is general agreement that the unresolved questions that face language researchers have nothing to do with the dialect’s linguistic validity, but instead concern the sociocultural conditions of its use [3]. Given current inequitable outcomes between African American students and their White peers, few if any of these questions are more important than those that bear on interactions between AAE and SCE in the classroom and other educational contexts. Which differences between African American English speaking children’s home and school dialects significantly impact tasks that like learning to read are critical to educational achievement, and what are the mechanisms by which they do so are important questions in need of answers.

With the goal of contributing to the literature that addresses these weighty questions, this paper reports the results of two experiments–an auditory Event Related Potential (ERP) study and an orally administered math test–both of which are part of a larger inquiry into how AAE speaking grade schoolers manage the differences between their home dialect and SCE. In keeping with the aim of identifying specific dialectal differences that affect educational achievement, both experiments focus on one particular difference argued to be especially consequential: as is held by a number of influential linguistic analyses (e.g., [4, 5]), we assume that unlike SAE, AAE has no verbal–*s*, the–*s* that, for example, turns *drink* into *drinks* in the SCE sentence *Mary drinks coffee* (see [6–8] for alternative views). There is mounting evidence that this seemingly small difference between the two dialects can negatively impact both AAE speaking children’s learning to read SCE [2] and their performance on orally administered mathematical reasoning tasks presented in that dialect [9, 10].

### Background

#### Motivating the math study

To date, reading achievement has been the primary focus of research into the role that differences in AAE and SCE grammar play in educational outcomes. Child language and literacy researchers have paid considerable attention to the general relationship between measures of children’s AAE use and other language and literacy measures (e.g., [11–17]). Many studies have shown a negative relationship between the two (e.g., [11, 14–16, 18, 19]), with a smaller number suggesting a positive relationship (e.g., [12, 20]). These mixed results help underscore the need to carefully examine the effect of specific divergences in grammar on specific educational tasks. There is no reason ahead of detailed investigation to expect all dialectal differences to have the same effect in all situations.

Collectively these studies suggest that at least some differences between home and school dialects, often referred to as dialect mismatches [3, 18], may negatively affect children’s academic achievement, and in the case of AAE, contribute to the disparity in reading performance between African American children and their White peers. For example, Terry and colleagues [14, 16] have shown that, generally speaking, non-mainstream dialect use is negatively predictive of early school-age children’s reading ability. A number of causal explanations have been suggested for this relationship. On one account, the need to separately store structures from both their home and school dialects leaves AAE speaking children with fewer cognitive resources available for understanding important aspects of classroom instruction, resulting in reading and other academic difficulties [3, 21]. Viewed this way, the principal issue is the cost, in cognitive terms, that children pay for switching between two dialects, a cost presumably paid even when the children have reasonable control over both language varieties. Although recent work on Korean/English bilinguals suggests highly proficient adult code-switchers incur little or no cost for language shifts [22], the case may very well be different for children. As an alternative to any price paid for code-switching, other researchers have proposed that it is AAE speaking children’s lack of familiarity with and control over mainstream structures and grammatical rules themselves that is the root of the problem. This lack of familiarity and control turning dialect mismatches into stumbling blocks to the acquisition and development of literacy skills [3, 14–16, 20, 23]. Of course, these two accounts are not mutually exclusive. Both may play a role, and individual children may face different challenges.

Washington, et al. (2013) [17] summarize the results of investigations into how speaking AAE at home affects children’s learning to read with the following observation: although from preschool through 5th grade AAE speaking students are more likely to struggle as readers than are their mainstream dialect speaking peers, not all children who speak higher levels of AAE are at-risk for experiencing reading failure. Who is, who is not, and why are questions still in need of answers, answers Washington and colleagues argue will only be found when we have a fuller account of the neurocognitive mechanisms underlying reading performance. They suggest computational modeling as one means to that end. Brown and colleagues (2015) [24] are among those who have used this technique to argue that children who use more AAE may, in general, be at greater risk for reading difficulties because differences in pronunciation make the task of learning to decode written-letter to-sound correspondences more complex than it is for speakers of more mainstream varieties of English [24].

The two experiments described herein are intended to move the current conversation beyond reading in that they point towards non-reading-specific mechanisms by which dialect mismatches may affect educational achievement. Labov and Baker (2010) [2] have argued that stumbling over verbal–*s* and possessive–*s* when asked to read aloud offers the clearest indication that an AAE speaking child is suffering from dialectal interference in reading. By investigating the role that verbal–*s* has on AAE speaking 2nd graders’ ability to answer orally presented math questions, the math experiment detailed in the current paper, side steps any difficulties children may have with written-letter to sound correspondences or any other reading-specific processes, thus leaving its results to implicate different, and likely more general, language processing mechanisms. Similarly, the auditory ERP experiment is designed not to shed light on neurocognitive mechanisms particular to reading, but on those engaged in verbally presented tasks, some of which are likely involved in the reading process as well. Together, the results of these experiments provide evidence that verbal–*s* can negatively affect AAE speaking 2nd graders classroom and test performance independent of reading.

Do differences in SAE and AAE grammar, in fact, affect children’s performance in class and on the tests that do so much to define their status in school? Framed this way–in terms of grammars–the question pushes researchers to be clear in their distinguishing of one grammar from another, a task that is not without its challenges. Two are particularly relevant to the current discussion. First, weather talking about morphology, syntax, phonology, or prosody, closely related dialects like AAE and SCE have far more in common than they do separating them, making it at times difficult to tell which dialect a speaker is using when. Second, individual dialects exhibit variability such that a speaker may use a feature unique to a particular dialect in one context and not in another without ever having left the dialect in question. Consider the AAE sentence *She making coffee*, translated into SCE as *She is making coffee*. Since Labov (1969) [4], the most widely accepted analysis of such sentences has been that they arise through a process of deletion. In line with Labov’s generalization that where mainstream varieties of English contract, AAE deletes, it is argued that *She is making coffee* becomes *She making coffee* in AAE through roughly the same process of phonological reduction that SCE speakers use to turn *She is making coffee* into *She’s making coffee*. The key point is that the sentence *She is making coffee*, is a part of both dialects. Although subject to different rules in the two different grammars, the copula *is* is a part of the morphological inventory of both AAE and SCE, and so the AAE speaking child who says *She is making coffee* is still operating within AAE grammar. Labov paints a very different picture for verbal–*s*. In contrast to the copula *is*, he argues that verbal–*s* is not a part of AAE. Green (2011) [25] draws the same conclusion for child AAE specifically. On this kind of account, the AAE speaking child who says *Mary makes coffee* is not speaking AAE. That child has either shifted from AAE grammar, where the sentence would be rendered *Mary make coffee*, into a more mainstream variety or at the very least mixed elements of two different systems. The distinction leads to a prediction: we expect different effects to stem from AAE speaking children’s encounters with morphology that is used variably in their home dialect than we do with morphology that is not a part of it.

This prediction is borne out in J. M. Terry et al. (2010, 2015) [9, 10], which present evidence that some, but not all, morphosyntactic differences between AAE and SCE can suppress 2nd grade AAE speaking children’s scores on the Woodcock-Johnson-R (WJ-R) Test of Applied Mathematics [26]. Examining the test scores of 75 students, this work employed a Bayesian Markov Chain Monte Carlo Method (MCMC) to estimate the effect of morphological mismatches between AAE and SCE in the WJ-R test questions, the presence of verbal–*s* being one. Consistent with the prediction above and Labov and Baker’s findings in the reading domain, possessive–*s* and verbal–*s*, the two features tested that are also argued to be absent from AAE grammar, were found to be the most important features in lowering overall scores. Both split the students into three groups: those highly affected, those moderately affected, and those who showed little effect of the features in question. For verbal–*s*, the MCMC model predicted that in the highly affected group (roughly 15% of the students), the average student would answer 9% more questions correctly if the effect of this feature were removed, suggesting an educationally significant impact, and leading directly to our predictions for the current math study: in a linguistically controlled test of addition and subtraction word problems, AAE speaking 2nd graders will perform better on questions that do not contain verbal–*s* than those that do.

#### Motivating the auditory ERP study

The aforementioned work on AAE makes psychological claims based on indirect and naturalistic evidence. In it, the effect verbal–*s* has on AAE speaking children is inferred from their responses to full sentences in which the morpheme is found, sentences spoken during familiar test taking activities. Building on the foundation this work provides, the auditory ERP experiment in the current study is designed to probe its claims more directly. A key strength of the ERP method is that it provides researchers with a real-time correlate of the brain’s electrophysiological activity as it responds to sensory and cognitive events. The technique’s high temporal resolution–its ability to take millisecond by millisecond recordings–makes it especially well-suited for the study of language processing as it progresses throughout time (for reviews, see e.g., [27–38]). In the current context, supplementing previous sentence-level behavioral results with ERP data offers the opportunity to support arguments that verbal–*s* can cause sentence processing difficulties for AAE speaking children with observations of the neurophysiological effect this morpheme has on them at the very time and place of its utterance. Further, collecting such data makes it possible to draw connections between verbal–*s*’ role in AAE grammar and specific processing mechanisms.

Important to the topic at hand–how AAE speaking children cope with SCE verbal–*s* morphology–three functionally distinct ERP effects related to processing syntactic and morphosyntactic anomalies have been identified in the literature. In response to word-level syntactic category errors, errors that disrupt local structure building, an Early Left Anterior Negativity (ELAN) occurs approximately 120–200 ms after the onset of the offending word (e.g., [39–48]). A different response is triggered by sub-word errors that preserve basic syntactic structure, but violate rules of tense, number or gender agreement. For instance, replacing the neuter article *das* with the masculine *den* in the German sentence *Sie bereist* *das* *Land auf einem kraftigen Kamel* (“She travels the_neuter_ land_neuter_ on a strong Camel”), results in a sentence that preserves the original’s basic structure, *Sie bereist* *den* *Land auf einem kraftigen Kamel*. However, the change introduces an agreement error as the neuter noun *land* requires a neuter article *das* (adapted from [49]). Errors of this type induce a Left Anterior Negativity (LAN) that occurs slightly later than the previously discussed ELAN, between approximately 300 and 500 ms post onset of the critical morpheme (e.g., [43, 50–57]). Both of these effects are typically followed by a late central-posterior positivity (P600) (for reviews, see e.g., [58–63]). This late positive deflection is often argued to result from either the reanalysis or the repair of structures initially assigned in error (e.g., [30, 35, 44, 45, 47, 62, 64–70]), both of these processes likely a part of some more general integration process required to complete sentence comprehension (e.g., [60, 62, 71–74]).

Each of the established ERP patterns described above (e.g., LAN followed by P600) results from familiar linguistic material being used in unfamiliar ways. However, a chief claim of this paper is that although AAE speaking children recognize SCE verbal–*s* as inflectional morphology, the morpheme itself is, in an important sense, unfamiliar to them. The linguistic landscape of the United States is such that all AAE speakers receive some exposure to verbal–*s*. Be that as it may, we contended that for many, especially children, this morpheme is not integrated into a tense and agreement system that they control, and is therefore less cognitively available to them than, say, past tense–*ed* or some other morpheme that is a part of AAE grammar. Thus, in encountering it, they face a different type of morphological error than investigated in the studies from which the reported patterns emerge–a type of error commonly encountered by non-mainstream dialect speaking children, but not directly addressed in this literature or the literature that investigates how second language learners cope with similarly misused morphology in their non-native language (cf. [75, 76]). These previous studies are nonetheless suggestive, providing a starting point for predicting AAE speaking children’s neurophysiological responses to different types of SCE sentences that use verbal–*s*.

**Fig 1:**
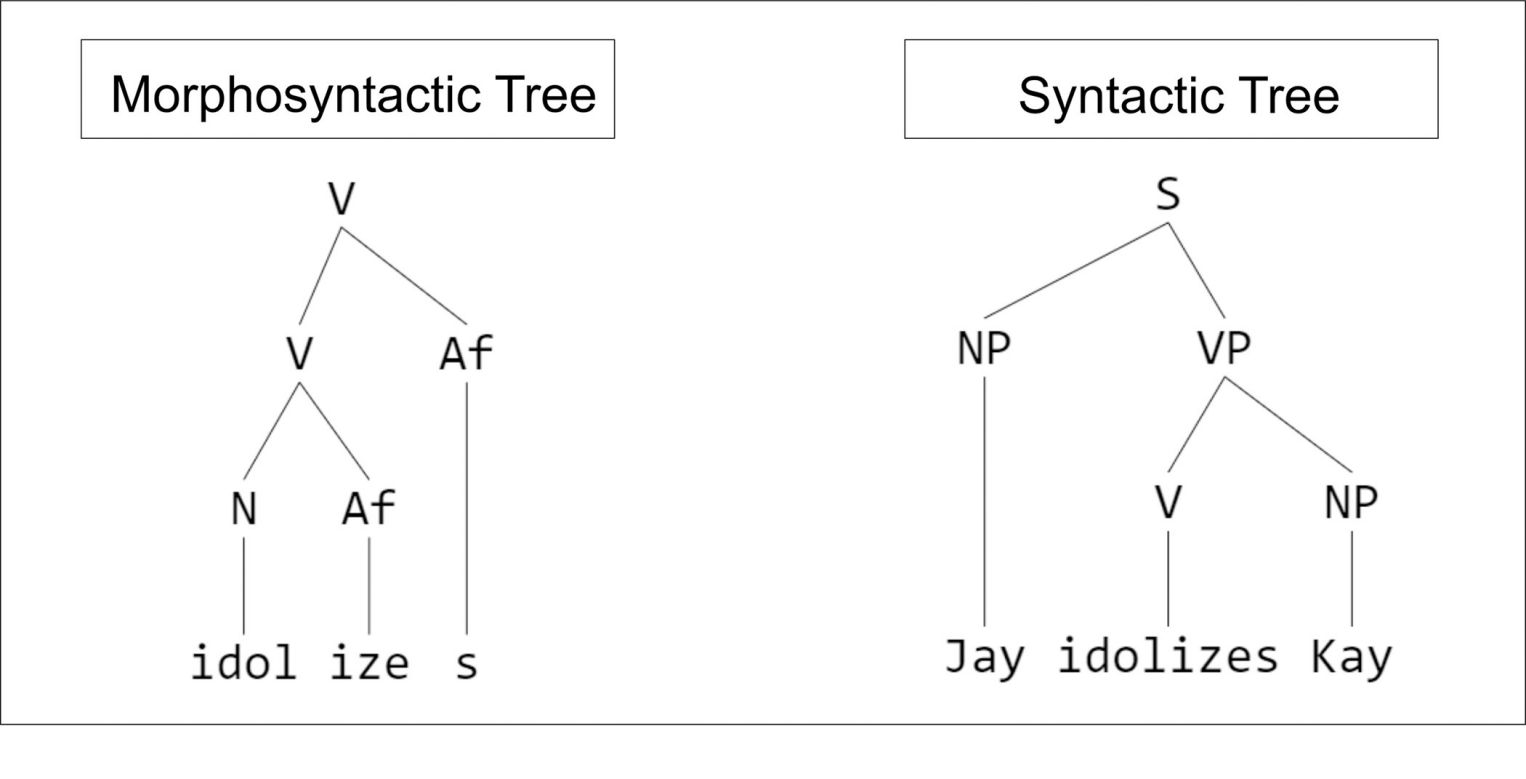
Syntactic and morphosyntactic similarity. The similarity of syntactic and morphosyntactic tree diagrams suggests that the two result from similar structure building operations. “Af” stands for Affix, “N” for Noun, “NP” for Noun Phrase, “V” for Verb, and “VP” for Verb Phrase.

Although still a matter of debate, data from both behavioral and neuroimaging studies suggest that regular inflectional morphemes like verbal–*s* are accessed separately from their stems, with stem and affix being fused together in a subsequent syntactic operation (for an overview, see e.g., [77]). Consequently, affixing verbal–*s* to the end of a verb can be viewed as a structure building operation similar to that of adding a new word to a syntactic structure. The similarity of the two tree structures in Fig 1 –one syntactic, the other morphosyntactic underscore the point. As discussed in greater detail in the section to follow, due to the lack of integration of the morpheme into their tense and agreement system, we expect AAE speaking children’s encounters with verbal–*s*, then, to signal a morphosyntactic structure building error and induce a response similar but not identical to the type triggered by phrase structure errors. Further, we expect verbal–*s* to be met with some measurable effect of the processing load the children incur as a result.

### The present experiments

The math test administered as a part of the current study consisted of 60 addition and subtraction word problems that varied in whether or not their prompts included verbs marked with SCE verbal–*s*. Other than the inclusion or exclusion of this one linguistic feature, the sentences were consistent with both AAE and SCE grammar. Similar to the results of previous experiments, the AAE speaking 2nd graders who participated in the study were predicted (and found) to have answered more problems correctly when the questions did not include verbal–*s*, and therefore were consistent with their home grammar.

Both the above prediction and those we make for the auditory ERP portion of the study stem from the assumption that, in the main, SCE verbal–*s* is less cognitively available to young AAE speakers than those morphemes that are a part of AAE grammar. As a part of the ERP experiment, participants listened to a total of 144 sentences that systematically varied in two ways: first, and most significantly, like the math experiment stimuli, they varied in whether or not their verbs were suffixed with SCE verbal–*s*. Second, they varied in whether their subjects were singular or plural, plural subjects being marked with the homophonous plural–*s*, a feature common to both SCE and AAE grammar.

Our expectations for the ERP data we collected were guided by both the ERP literature and the same behavioral findings that influenced our math test predictions above. As previously noted, we expected AAE speaking children to treat the presence of the verbal–*s* morpheme in singular subject sentences like *Mary makes coffee* as a morphosyntactic structure building error similar but not identical to a phrase structure building error. Thus, taking established ERP patterns as a starting point, we expected our study participants to respond to verbal–*s* in such sentences with an ELAN, albeit one distinct from both the kind of negativity triggered by phrase structure violations and the kind triggered by agreement errors. In the behavioral realm, J. M. Terry et al. (2010) [9] have argued that the propensity for verbal–*s* to depresses AAE speaking 2nd graders’ scores on verbally administered tests of math word problems is the result of the children’s bearing an increased working memory load itself due to their attempts to process the less than fully available morpheme. Previous ERP studies have associated such increases in working memory load with long lasting negativities (see e.g., [78]). Together with the previous prediction, this line of reasoning leads to another: that in addition to producing an ELAN indicative of a morphosyntactic structure building problem, the AAE speaking children in the present study would respond to verbal–*s* with a separate long lasting negativity indicative of the resulting increase in working memory load, the one effect following the other.

The crossing of singular subject vs. plural subject and presence vs. non-presence of verbal–*s* in the design of the ERP experiment allowed for the testing of another hypothesis: that young AAE speakers could be “tricked” into interpreting SCE verbal–*s* as a misplaced plural–*s*. Although ungrammatical in both SCE and AAE, we expected plural subject–*s* marked sentences like *The workers drinks coffee* to do just that, the salience of the plural marker combined with children’s lack of full access to verbal–*s* causing the confusion. Misidentified as a plural, we expected that participants would treat verbal–*s* as familiar, fully accessible morphology that had been used incorrectly. Thus, in line with previously established ERP patterns, we expected the ungrammatical verbal–*s* to induce a LAN-like effect followed by a P600 or some other late positive effect, the trailing ERP effect reflecting the repair or integration cost of the misidentified morpheme.

The following sums up our ERP predictions: assume that ERPs are timelocked to the critical verbal–*s* morpheme. Compared to *The worker drink coffee* (singular subject, no–*s* on the verb), a sentence like *The worker drinks coffee* (singular subject,–*s* marked verb) should elicit an early negativity, followed by a prolonged negativity, both pronounced at the anterior site. Compared to *The workers drink coffee* (plural subject, no–*s* on the verb), a sentence like *The workers drinks coffee* (plural subject,–*s* marked verb) should elicit an early anterior negativity and a late central-posterior positivity. The topographical distribution of these ERP effects may differ from that found in previous studies that tested adult participants, as compared to adults, children often recruit additional cognitive resources when they engage in the same psycholinguistic processing tasks (for an overview, see e.g., [79, 80]).

### Auditory ERP experiment

This section details the auditory ERP experiment portion of the study outlined above. The study was approved by the IRB and Office of Human Research Ethics at the University of North Carolina at Chapel Hill (Study Number 13–1565). All the study participants were children. Written consent was obtained from the participants’ guardians, and oral assent from the participants themselves.

### Methods

#### Participants

Thirty-two AAE speaking 2nd graders participated in the auditory ERP experiment described below. The data for twenty-two participants (10 male and 12 female; mean age 7.55 years and 6.32 months) were included in the final data analysis. The remaining ten participants either did not complete the experiment (six participants) or had their data excluded from final analysis due to excessive EEG artifacts (four participants). All participants were right-handed, had normal or corrected-to-normal vision, and were free of any known neurological or hearing disorders.

The dialect-speaking status of each participant was supported by results from the Diagnostic Evaluation of Language Variation (DELV) Screening Test [81] and a modified version of the Third Person Present Tense subtest of the Wiig Criterion Referenced Inventory of Language (Wiig CRIL) [82]. Twenty-one of the final twenty-two participants’ screening test results fell into the DELV category of “strong variation from Mainstream American English”, supporting the conclusion that they were indeed speakers of AAE. (We treat Mainstream American English here as a synonym for SCE.) The remaining one participant was identified by the DELV as having “some variation from Mainstream American English”, again suggesting that this participant was an AAE speaker. No participants fell into the DELV category of Mainstream American English speaker.

In contrast to the DELV screener’s more global evaluation of dialect status, the CRIL subtest was used specifically to measure the participants’ production of verbal–*s*. The verbal–*s* morpheme is a part of SCE but not AAE grammar; thus, it follows that low levels of its production are consistent with AAE use. We assume that when a child whose primary dialect is AAE does produce verbal–*s*, that production reflects either emerging SCE or code-switching/mixing on the part of the child. Because the goal in administering the CRIL subtest was to assist in evaluating participants’ dialect-speaking status, as opposed to their ability to produce an unfamiliar grammatical pattern, we modified the test’s instructions so that when modeling answers in the demonstration, the test administrator only used sentences that paired a plural subject with a suffix-less verb, sentences like “The ballerinas dance”. As a result, no sentence used to model a potential test answer ever contained verbal–*s*, and all model answers were consistent with both AAE and SCE grammar. No other modifications to the test were made. The CRIL subtest consisted of a total of 10 items. The mean percentage of verbal–*s* use on this test and its SD were 39.09 ± 33.08%. Anecdotal evidence suggests that the large standard of deviation here is due, at least in part, to the classroom-test-like format of the CRIL subtest coupled with participants’ awareness that classroom teachers expect students to use verbal–*s*. When asked to repeat an answer so that it could be better heard, one participant remarked “Oh, do you want me to put that–*s* on it?” The test administrator remained neutral in response and asked him to simply do his best to answer. Further, we note that the one participant the DELV identified as having only “some variation from Mainstream American English” scored 20%, for the use of verbal–*s* on the CRIL subtest (well below SCE mastery levels), providing additional evidence that this participant was indeed an AAE speaker.

#### Stimuli

As exemplified by the sentence “One kid from Swan Village eats chocolate cake”, test sentences for the auditory ERP experiment fit the following sentential frame: NUMBER kid(s) from NAME Village VERB OBJECT. Following a 2 x 2 factorial design, the stimuli varied first in whether the subject noun phrase (NP) was the singular “One kid” (conditions SS and SN, the leading “S” standing for singular) or the plural “Six kids” (conditions PS and PN, the leading “P” standing for plural). Second, the stimuli varied in whether the sentence’s verb carried an–*s* suffix (SS and PS conditions, the trailing “S” standing for suffixed) or did not carry an–*s* suffix (SN and PN conditions, the trailing “N” standing for no suffix). Example sentences for each condition are provided in Table 1. Condition SS (a singular subject NP paired with a verb with–*s*) is consistent with SCE, but not AAE grammar. Condition SN (a singular subject NP paired with a verb without–*s*) is consistent with AAE, but not SCE grammar. Condition PS (a plural subject NP paired with a verb–*s*) is not consistent with either SCE or AAE grammar, while condition PN (a plural subject NP paired with a verb without–*s*) is consistent with both grammars. Thirty-six sets of test sentences were created, each set containing four sentences (one sentence per condition), for a total of 144 sentences. The test sentences were all spoken by a bidialectal African American woman, who at the time they were recorded, had been a life-long member of the community from which the participants were drawn.

**Table 1:** Example stimuli for the four experimental conditions tested in the auditory ERP experiment and their grammatical statuses in AAE and SCE grammar.

Condition	Label	Example Sentence	Status in AAE	Status in SCE
**Singular Subject NP,–*s* on Verb**	**SS**	One kid from Swan Village eats chocolate cake.	Ungrammatical	Grammatical
**Singular Subject NP, No–*s* on Verb**	**SN**	One kid from Swan Village eat chocolate cake.	Grammatical	Ungrammatical
**Plural Subject NP,*–s* on Verb**	**PS**	Six kids from Swan Village eats chocolate cake.	Ungrammatical	Ungrammatical
**Plural Subject NP, No–*s* on Verb**	**PN**	Six kids from Swan Village eat chocolate cake.	Grammatical	Grammatical

To monitor participants’ attention throughout this ERP portion of the experiment, a behavioral task was introduced into the experimental design. Each ERP test sentence was matched with a “comparison sentence” for which no ERP data were analyzed. These sentences were spoken by a bidialectal African American man, who although not a member himself, has close ties to the participants’ community through family and friends. The resulting test-comparison sentence pairs served as stimuli for the behavioral task, a sentence discrimination task in which participants were asked to indicate whether or not the two sentences in a given pair were identical. Of the 144 comparison sentences created, half matched their corresponding test sentences word for word. The other half differed from their corresponding test sentences by one word, changing either the number of kids, village name, verb or the verb’s object from test sentence to comparison sentence. The sentences always differed such that these words could not be misinterpreted as being semantically equivalent. For example, “One kid from Swan Village bakes chocolate cake” would serve as a suitable comparison sentence for the test sentence “One kid from Swan Village eats chocolate cake”. Given the pair, participants would be expected to recognize that these two sentences are different due to their different verbs, *bakes* and *eats*. The test sentence-comparison sentence differences were evenly distributed across the manipulated parts of the test sentence. The condition of the test sentence-comparison sentence pair is defined by the test-sentence condition.

Based on Thomas (2007, 2010, 2015) [83–85], the segmental and prosodic features of the stimuli (both the test and comparison sentences) were controlled to avoid any dialectical bias in their phonology. Stimuli sentences were constructed so that they only contained segments pronounced the same in African American and more mainstream varieties of English, and speakers were instructed in how to control their prosody to avoid ethnically identifiable patterns. Thus, while the sentences may have retained some phonological characteristics reflective of geographic region, they were designed to be phonologically consistent with both AAE as spoken by the study participants and other more mainstream varieties of Southern American English spoken in the area. All sentences were digitally recorded in stereo at a 16-bit/44.1-kHz sampling rate.

**Fig 2:**
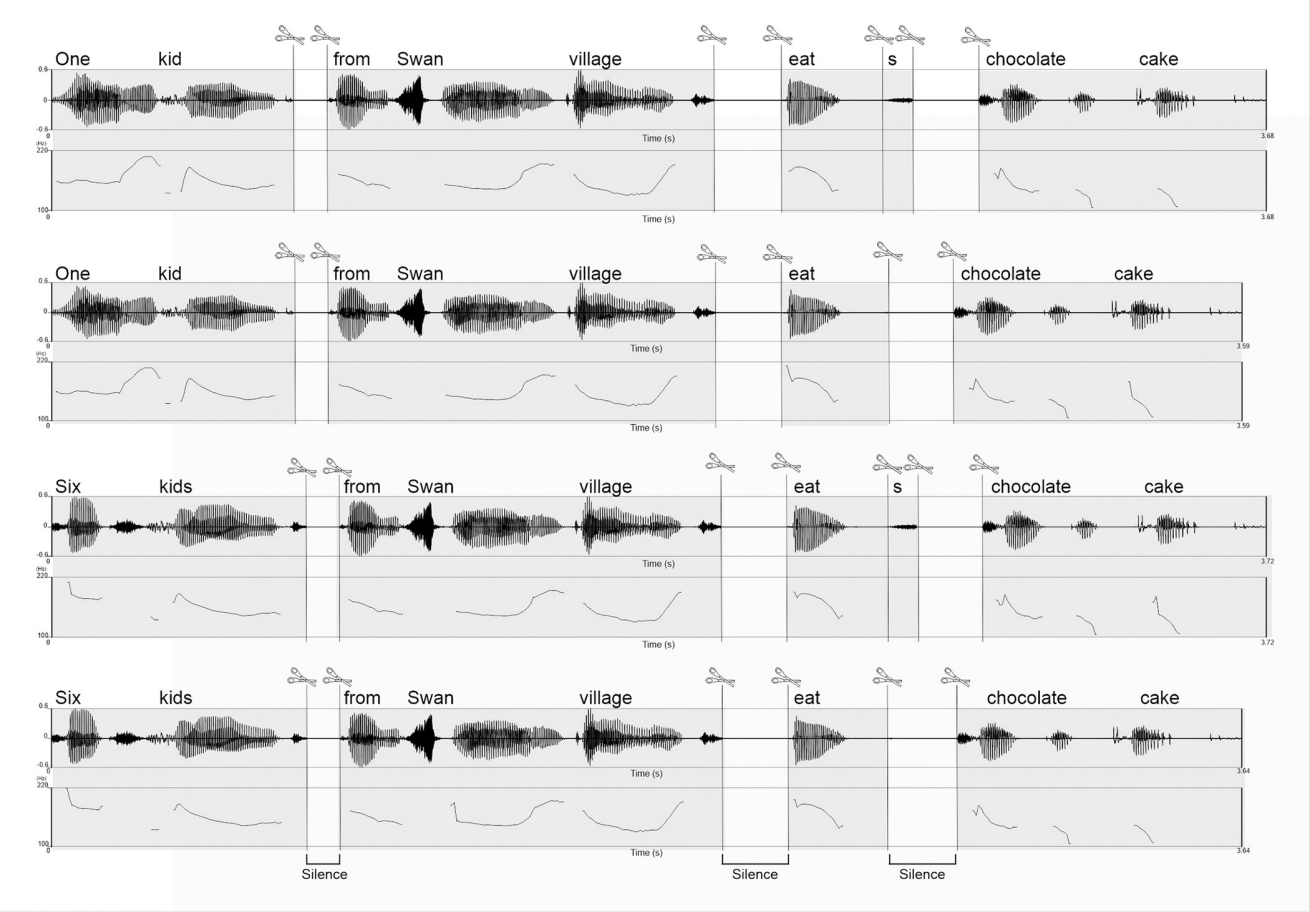
Example pitchtracks and illustration of the splicing procedure applied to the four experimental conditions tested in the auditory ERP experiment. Scissors indicate the positions where auditory files were appended. A 100 ms period of silence was inserted after “one kid” and “six kids”, and a 200 ms period of silence was inserted after “from Swan village” and “eat(s)”.

Audio files for the test sentences were constructed using a splicing (i.e., audio “copy and paste”) procedure to ensure that across all four test conditions and across all sentences within those conditions, instances of the same words were acoustically identical. Similarly, a single audio recording of the verbal–*s* morpheme was created, copied, and then spliced onto the verbs on which the morpheme occurred, thus guaranteeing that all instances of this critical feature were also acoustically identical. Once put together in this manner, each test sentence audio file was reviewed to make sure that it maintained the qualities of natural speech. Fig 2 presents pitchtracks for the example test sentences given in Table 1 along with indications of where the sentences were spliced. Tables 2 and 3 present the mean f0 maxima and duration of each phrase for the test stimuli. All stimuli were normalized to an intensity level of 70 dB. All audio editing was done using Adobe Audition CS6 (San Jose, CA). Acoustic measurements of the stimuli were taken with Praat [86].

**Table 2:** Mean max pitch (Hz) for each phrase of the sentences tested in the auditory ERP experiment with their SDs (in parentheses).

		Mean Max Pitch (Hz)			
Condition	Label	Subject NP	Prepositional Phrase	Verb	Object NP
**Singular Subject NP,–*s* on Verb**	**SS**	204.81	185.94 (9.15)	185.88 (33.01)	217.48 (99.98)
**Singular Subject NP, No–*s* on Verb**	**SN**	204.81	185.94 (9.15)	185.88 (33.01)	217.48 (99.98)
**Plural Subject NP,*–s* on Verb**	**PS**	204.81	185.94 (9.15)	185.88 (33.01)	217.48 (99.98)
**Plural Subject NP, No–*s* on Verb**	**PN**	204.81	185.94 (9.15)	185.88 (33.01)	217.48 (99.98)

Note: The splicing procedure used to create the stimuli ensured that except for the phrases where systematic manipulations (whether the subject NP was singular or plural, and whether or not the verb carried an–*s* suffix) were applied, the pitch of the phrases remained the same across all conditions.

**Table 3:** Mean duration (ms) for each phrase of the sentences tested in the auditory ERP experiment with their SDs (in parentheses).

		Mean Duration (ms)			
Condition	Label	Subject NP	PrepositionalPhrase	Verb	Object NP
**Singular Subject NP,–*s* on Verb**	**SS**	733.52	1078.00 (85.10)	462.40 (76.70)	830.40 (71.80)
**Singular Subject NP, No–*s* on Verb**	**SN**	733.52	1078.00 (85.10)	369.80 (94.40)	830.40 (71.80)
**Plural Subject NP,*–s* on Verb**	**PS**	790.7	1078.00 (85.10)	462.40 (76.70)	830.40 (71.80)
**Plural Subject NP, No–*s* on Verb**	**PN**	790.7	1078.00 (85.10)	369.80 (94.40)	830.40 (71.80)

Note: The splicing procedure used to create the stimuli ensured that except for the phrases where systematic manipulations (whether the subject NP was singular or plural, and whether or not the verb carried an–*s* suffix) were applied, the duration of the phrases remained the same across all conditions.

To make the task more engaging for the participants, all of whom were children, the auditory stimuli described above were embedded inside animated video clips so that the recorded sentences appeared to be spoken by two animal-like cartoon characters. After a female cartoon character spoke a test sentence, a male character would speak a comparison sentence. A separate video clip was prepared for each of the stimulus sentence pairs (144 video clips in total). The GoAnimate™ (now Vyond™) software package (GoAnimate, Inc, San Mateo, CA) was used to create the animated video clips (for more details, see Procedures).

#### Procedures

All ERP experimental sessions took place in a quiet room at the participants’ elementary school. Each participant individually completed one approximately one hour-long ERP session, separate from all other testing, which took place on different days. Participants were randomly assigned to one of four counterbalanced lists of stimuli, with each list divided into eight experimental blocks, each block containing 18 test sentences. The stimuli within each block were pseudorandomized so that no two sentences of the same condition ever appeared in more than two trials per sequence.

At the start of each session, a participant was seated in a chair placed approximately 100 cm away from the computer screen and loudspeakers that played the video clip and auditory stimuli. Before the stimuli were presented, participants were told that they would be listening to a series of paired sentences spoken by two cartoon characters. They were instructed to listen to each sentence and indicate whether the two sentences were identical. Each participant heard five pairs of practice sentences before the eight experimental blocks began. At the end of each block, participants were asked to take a short break. However, they were also advised that they could request a break at any time during the experiment.

**Fig 3:**
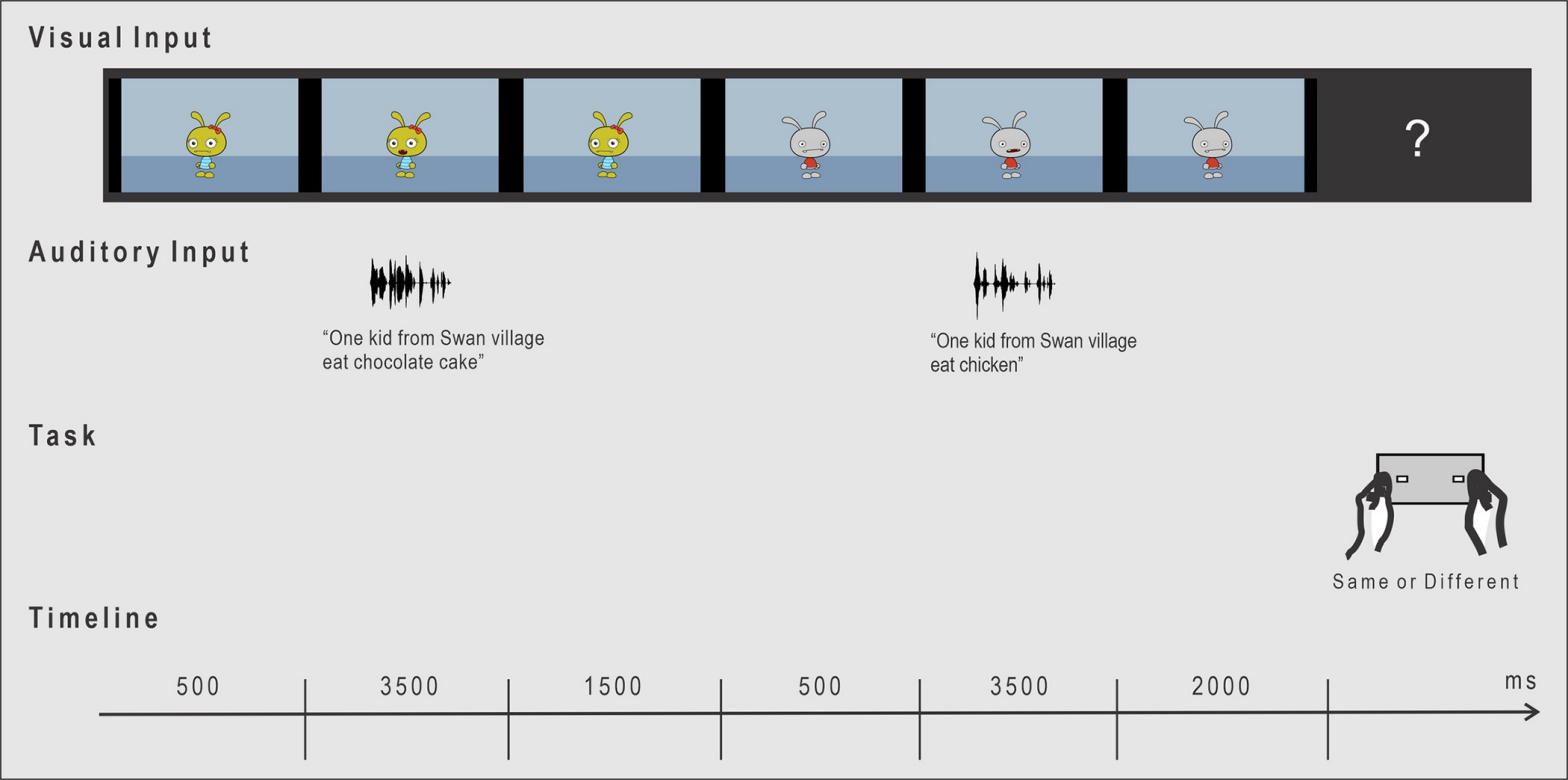
Trial sequence in the auditory ERP experiment. The appearance of the female cartoon character on the computer screen marked the beginning of each trial. After a participant gave his or her response to the task, a blank screen appeared for 2000 ms before the next trial started.

At the beginning of each trial within a block, the female character appeared in the middle of the computer screen (see Fig 3). 500 ms after her appearance she spoke a test sentence approximately 3500 ms in length (see Table 3). After completing the sentence, she remained on screen for 1500 ms before disappearing and being replaced by the male character. 500 ms after his appearance, the male character then spoke a comparison sentence. 3500 ms after doing so, he disappeared and 2000 ms later, a question mark appeared on the screen. The question mark remained on screen until either the participant pressed one of two buttons on a response pad to indicate whether or not the sentences the cartoon characters spoke were identical, or until a 5000 ms time-out interval passed. No feedback was given to participants after they registered their responses. An inter-trial interval of 2000 ms during which a blank screen was shown occurred before the next trial began.

*Electrophysiological recording and preprocessing*. During the presentation of the test sentences, electroencephalograms (EEG) were recorded from 32 Ag/AgCl scalp electrodes via an EASYCAP EEG recording cap (Brain Vision, Herrsching, Germany). In compliance with the standard 10–20 system for electrode positioning (cf. [87]), EEG signals were recorded from the following scalp positions: F1/F2, F3/4, F7/8, FC1/2, FC5/6, C3/4, T7/8, CP1/2, CP5/6, TP9/10, P3/4, P7/8, PO3/4, O1/2, FZ, CZ, PZ, and OZ, with the FPZ channel serving as the ground electrode. To control for artifacts resulting from ocular movements, the FP1 and FP2 channels were used to record electrooculograms (EOG). All scalp electrodes were referenced online to the left mastoid and re-referenced offline to linked mastoids. Electrode impedances were kept below 10 kΩ. EEG and EOG channels were amplified using an actiCHamp amplifier (Brain Vision, Herrsching, Germany). Both EEG and EOG were continuously recorded at a sampling rate of 1000 Hz. To remove slow signal drifts, EEG data were filtered with a 0.3–20 Hz band pass filter. Trials contaminated by EOG saturation, amplifier saturation, or other artifacts were identified by a semi-automatic artifact rejection using an automatic criterion of 40 μV standard deviations (calculated in 200 ms sliding windows) and were manually checked afterwards. Trials containing artifacts (39.71% of all trials) were eliminated from further analyses. Likewise, the data points of participants with fewer than 18 trials in one or more than one condition (data points of four participants) were not analyzed.

*Data analysis procedures*. Data for the behavioral task–the sentence discrimination task used to monitor participants’ attention during the collection of ERP data–were analyzed as follows: incorrect response rates and reaction times were calculated for each of the four experimental conditions. A response was considered incorrect if it was either false (i.e., the wrong answer) or was supplied after the time allotted to answer had elapsed. Incorrect responses were not included in the reaction time analysis. To investigate the effect of experimental condition on incorrect response rate and reaction time, separate one-way ANOVAs were carried out using the factor CONDITION (SS, SN, PS, PN) and error terms based on participant (*F*_*1*_) and item variability (*F*_2_). Following the one-way ANOVAs, two critical pairwise comparisons of the conditions were performed: SS vs. SN and PS vs. PN. In addition, contrast tests were conducted where appropriate.

All EEG data for test sentences were analyzed regardless of participants responses in the behavioral task. ERPs were time-locked to the onset of the end of the verb, the position where verbal–*s* attaches in SCE grammar, and average ERPs were computed for a time epoch that ranged from 0 ms to 1500 ms beginning from that target position (see Table 1). For each electrode, ERPs were averaged per participant per condition before grand averages were calculated across all participants across all conditions. On the basis of visual inspection, two time windows (200–400 ms and 400–900 ms) were chosen for statistical analysis. For each of these time windows, two critical pairwise comparisons were made to test our specific predictions: SS vs. SN and PS vs. PN. To examine the effect of experimental condition on the ERP signatures within each time window, prior to these condition comparisons, a repeated measures 4 x 3 x 2 Global ANOVA was performed with the experimental variable of COND (SS, SN, PS, PN) and the following topographical variables: REG (Anterior, Central, Posterior) and HEM (Left, Right). For the lateral electrodes, the topographical variables (REG and HEM) were fully crossed yielding six Regions Of Interest (ROIs); left anterior (F3, F7, FC1, FC5), right anterior (F4, F8, FC2, FC6), left central (C3, T7, CP1, CP5), right central (C4, T8, CP2, CP6), left posterior (P3, P7, PO3, O1), and right posterior (P4, P8, PO4, O2). (For the complete results of the repeated measures 4 x 3 x 2 Global ANOVA, see S1 and S2 Figs). In addition, to examine the effect of the presence or absence of verbal–*s* and its interaction with the subject NP while keeping other factors constant, a repeated measures 2 x 2 x 3 x 2 Global ANOVA was also conducted for each of the two time windows. In the repeated measures 2 x 2 x 3 x 2 Global ANOVA, the experimental variable was treated as a 2 x 2 factorial design, with the first variable VERB (SS, PS vs. SN, PN, i.e.,–*s* on verb vs. No–*s* on verb) and the second variable SUBJ (SS, SN vs. PS, PN, i.e., singular subject NP vs. plural subject NP). The topographical variables REG and HEM of the 2 x 2 x 3 x 2 Global ANOVA remained the same as the 4 x 3 x 2 Global ANOVA. Midline electrode analyses for the two Global ANOVAs (4 x 3 x 2 Global ANOVA and 2 x 2 x 3 x 2 Global ANOVA) are not reported below, however, they yielded results comparable to those obtained in the lateral electrodes. Statistical analyses were carried out in a hierarchical manner, i.e., only significant interactions (*P* < 0.05) were resolved. Whenever a variable included more than one degree of freedom in the numerator, corrected values were reported [88]. Effects attributed to the topographical variables alone (i.e., a main effect of REG and HEM or an interaction between two topographical variables, REG x HEM) are not reported. Only significant effects (*P* < 0.05) are reported.

### Results

#### Behavioral data

Table 4 presents mean correct response percentages and mean response times for each condition of the behavioral task. Overall, performance on the task was high (mean correct response across all conditions 80.05%), suggesting that participants paid sufficient enough attention to the sentences in this behavioral task for the ERP data collected at the same time to be meaningfully analyzed. Statistical analyses show that participants performed best when the test sentence-comparison sentence pair being evaluated was in the SS condition, that is, when the two sentences participants were comparing both made use of a singular subject paired with verbal–*s*. Specifically, a one-way ANOVA showed that this effect of CONDITION on correct response was significant for the participant analysis (*F*_*1*_(3,63) = 3.68, *P* < 0.05, *F*_*2*_(3,105) = 2.06, *P* = 0.11). The significant result stemmed from participants’ performing better on the SS condition than the other two conditions (SN and PS). Pairwise comparisons showed significantly better performance in the SS condition than the SN and PS conditions in the participant analysis (SS vs. SN: *F*_*1*_(1,21) = 11.44, *P* < 0.005, *F*_*2*_(1,35) = 2.74, *P* = 0.11; SS vs. PS: *F*_*1*_(1,21) = 15.71, *P* < 0.001, *F*_*2*_(1,35) = 8.09, *P* < 0.01; SS vs. PN did not reach significance: *F*’s < 4.10). No other pairwise comparisons produced significant results (*F*’s < 1.00). A contrast test comparing SS and the mean of the other three conditions (SN, PS, and PN) was also conducted. Consistent with those of the pairwise tests, the results of this test indicated that SS yielded significantly better performance than the mean of all other conditions in the participant analysis (*F*_*1*_(1,21) = 16.30, *P* < 0.001, *F*_*2*_(1,35) = 0.15, *P* = 0.71). It is notable that SS, one of the two sentence types that are ungrammatical in AAE, and of them, the only one that is grammatical in SCE, gave rise to the strongest performance by the AAE speaking 2nd graders on this task. These results likely suggest that the combination of the ungrammaticality of the sentences and the fact that this construction is taught in school garnered the participants’ attention in a way that was beneficial to the task. For completeness, statistical analyses were carried out for the response time data. No significant results were found (*F*’s < 1.85).

**Table 4:** Mean percent correct response (%), response times (ms), and their SDs (in parentheses) for each condition in the behavioral task for the auditory ERP experiment.

Condition	Label	Correct Response (%)	Response Time (ms)
**Singular Subject NP,–*s* on Verb**	**SS**	83.78 (8.69)	892.01 (301.04)
**Singular Subject NP, No–*s* on Verb**	**SN**	79.23 (9.22)	928.51 (321.26)
**Plural Subject NP,*–s* on Verb**	**PS**	78.36 (10.23)	974.56 (381.00)
**Plural Subject NP, No–*s* on Verb**	**PN**	78.84 (10.32)	907.69 (301.99)

#### ERP data

**Fig 4:**
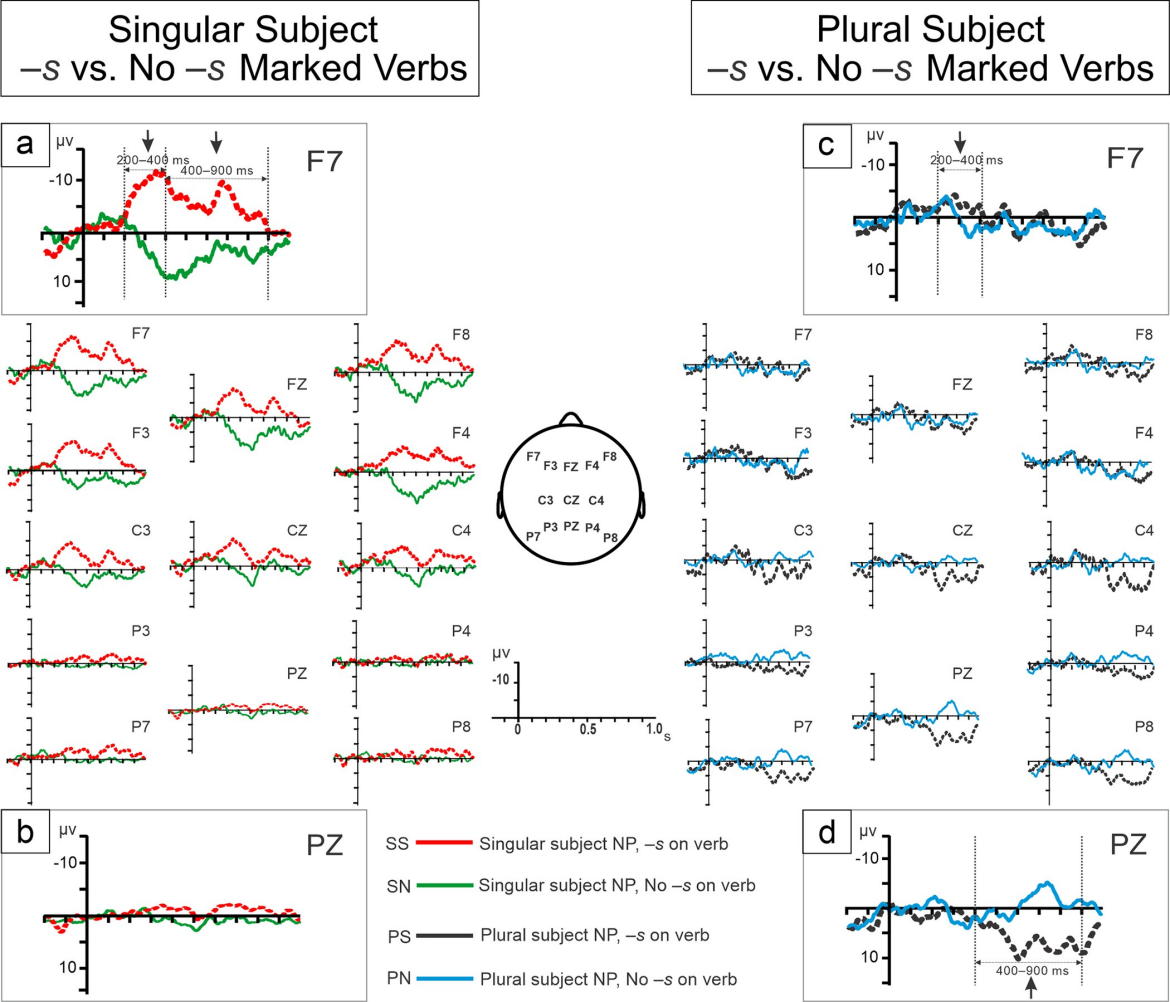
Grand average ERPs for the four experimental conditions. ERPs were time-locked to the onset of the target position, i.e., at the right edge of the underlined word in Table 1. Negativity is plotted upwards. Left plot (SS vs. SN): Compared to AAE grammatical sentences that pair a singular NP subject with a suffix-less verb (green), AAE ungrammatical sentences with singular NP subjects and–*s* marked verbs (red) showed a bilateral anterior-central negativity in both the 200–400 ms and 400–900 ms time windows (Zoom-in plots (a) and (b)). Right plot (PS vs. PN): Compared to AAE grammatical sentences that pair a plural subject NP with a suffix-less verb (blue), AAE ungrammatical sentences with plural subject NPs and–*s* marked verbs (black) showed a bilateral anterior negativity (200–400 ms), followed by a bilateral central-posterior positivity (400–900 ms) (Zoom-in plots (c) and (d)).

Fig 4 visually depicts the grand averaged ERPs for each of the four experimental conditions given in Table 1. Each ERP was time-locked to its target position–the right edge of its sentence’s verb–as indicated in that same table. Tables 5 and 6 give the ERPs’ mean values and SDs in microvolts for the two time windows described in the Methods section (200–400 ms and 400–900 ms). Visual inspection of the data revealed the following patterns. First, compared to those sentences that paired a singular subject NP with a suffix-less verb (SN, Grammatical in AAE), sentences that contained a singular subject with an–*s* marked verb (SS, Ungrammatical in AAE) elicited a bilateral anterior-central negativity in both 200–400 ms and 400–900 ms post the target position. Second, compared to plural subject sentences with suffix-less verbs (PN, Grammatical in AAE), plural subject sentences that contained–*s* marked verbs (PS, Ungrammatical in AAE) showed a bilateral early anterior negativity from 200 to 400 ms, followed by a bilateral late central-posterior positivity that lasted from 400 to 900 ms. These data patterns are supported by the statistical analyses that follow.

**Table 5:** Mean ERP amplitudes (μV) and their SDs for the four experimental conditions in the 200–400 ms time window.

		Time Windows (Time-locked to Target Onset)					
		200–400 ms					
		Anterior		Central		Posterior	
Condition	Label	Mean	SD	Mean	SD	Mean	SD
**Singular Subject NP,–*s* on Verb**	**SS**	-8.59	5.84	-4.97	3.17	-0.81	3.06
**Singular Subject NP, No–*s* on Verb**	**SN**	1.17	4.88	-0.91	3.54	-1.49	3.92
**Plural Subject NP,*–s* on Verb**	**PS**	-4.29	8.65	-2.56	5.94	-0.57	6.01
**Plural Subject NP, No–*s* on Verb**	**PN**	0.82	6.46	-0.01	3.32	-1.00	5.23

Note: Values are presented for each of the three lateral regions, Anterior (averaged over F3, F4, F7, F8, FC1, FC2, FC5, and FC6), Central (C3, C4, T7, T8, CP1, CP2, CP5, and CP6), and Posterior (P3, P4, P7, P8, PO3, PO4, O1, and O2).

**Table 6:** Mean ERP amplitudes (μV) and their SDs for the four experimental conditions in the 400–900 ms time window.

		Time Windows (Time-locked to Target Onset)					
		400–900 ms					
		Anterior		Central		Posterior	
Condition	Label	Mean	SD	Mean	SD	Mean	SD
**Singular Subject NP,–*s* on Verb**	**SS**	-6.26	8.64	-3.29	4.62	-1.35	3.79
**Singular Subject NP, No–*s* on Verb**	**SN**	2.69	4.95	0.78	3.46	-0.23	3.22
**Plural Subject NP,*–s* on Verb**	**PS**	1.43	8.7	1.71	4.5	3.65	3.86
**Plural Subject NP, No–*s* on Verb**	**PN**	2.08	5.36	-0.43	3.29	-2.28	4.62

Note: Values are presented for each of the three lateral regions, Anterior (averaged over F3, F4, F7, F8, FC1, FC2, FC5, and FC6), Central (C3, C4, T7, T8, CP1, CP2, CP5, and CP6), and Posterior (P3, P4, P7, P8, PO3, PO4, O1, and O2).

*Results of critical pairwise comparisons of the experimental conditions*. Table 7 presents the statistical results of the critical condition comparisons. As predicted, the comparison of single subject sentences with and without verbal–*s* (SS vs. SN) and the comparison of plural subject sentences with and without verbal–*s* (PS vs. PN) were both significant in the 200–400 ms and 400–900 time windows. More precisely, the comparison SS vs. SN was significant in both the anterior and central regions in both the 200–400 ms and 400–900 ms time windows. The comparison PN vs. PS reached significance in both the anterior and central regions in the 200–400 ms time window and in the central and posterior regions in the 400–900 ms time window. In addition, comparing SS and PS showed that SS was significantly more negative than PS in the anterior region in the 200–400 ms time window (*F*(1,21) = 4.84, *P* < 0.05). Together, the results of these comparisons support the data pattern observed via visual inspection. (See S1 and S2 Figs for the results of the complete statistical analyses of the ERP data).

**Table 7:** Results of critical pairwise comparisons.

			Time Windows (Time-locked to Target Onset)			
			200–400 ms		400–900 ms	
*Comparison*	REGs	*df*	*F*	*P*	*F*	*P*
***SS vs*. *SN***	**All**	1,21	39.02	< 0.0001	21.53	< 0.0005
	**Anterior**	1,21	84.10	< 0.0001	26.19	< 0.0001
	**Central**	1,21	25.10	< 0.0001	14.28	< 0.005
	**Posterior**	1,21	0.59	n.s.	1.32	n.s.
***PS vs*. *PN***	**All**	1,21	5.60	< 0.05	11.63	< 0.005
	**Anterior**	1,21	11.55	< 0.005	0.15	n.s.
	**Central**	1,21	6.93	< 0.05	9.70	< 0.01
	**Posterior**	1,21	0.05	n.s.	24.69	< 0.0001

Note: Pairwise comparisons between SS and SN and between PS and PN were performed across all regions (see the rows for All in the table) as well as each region, i.e., REG (Anterior, Central, Posterior). For the four experimental conditions and their labels, refer to Table 1.

*2 x 2 x 3 x 2 Global ANOVA*. In line with the study hypothesis, the statistical analyses presented above showed a difference in participants’ ERP responses to those sentences that contained a verbal–*s*, ungrammatical in AAE, and those that did not, grammatical in AAE. Still, rather than stemming from anything to do with grammar, it is possible that this difference merely reflects the differences in the test sentences’ lengths and acoustic properties that were introduced by the presence or absence of the–*s* marker itself. Unlike grammatical accounts, however, this explanation does not predict significant interactions between the presence or absence of the–*s* marker and other grammatical characteristics of the test sentences. To test whether such interactions occurred, as described in the Methods section, a 2 x 2 x 3 x 2 Global ANOVA with the first factor VERB (*–s* marker on the verb, No–*s* marker on the verb), the second factor SUBJ (Singular subject NP, Plural subject NP), the third factor REG (Anterior, Central, Posterior), and the fourth factor HEM (Left, Right), was performed for both selected time windows. The results of these analyses support both the ERP effects reported so far and the more grammatical account of them. A significant VERB x SUBJ interaction was found for all of the critical analyses. The 2 x 2 x 3 x 2 Global ANOVA showed a significant interaction of VERB x SUBJ in both selected time windows (see Table 8). (For completeness, all significant interactions found in the 2 x 2 x 3 x 2 Global ANOVA were resolved. For the results, see S3 Fig.)

**Table 8:** Results of 2 x 2 x 3 x 2 Global ANOVA for the two selected time windows (200–400 ms and 400–900 ms).

		Time Windows (Time-locked to Target Onset)			
		200–400 ms		400–900 ms	
***Effect***	***df***	***F***	***P***	***F***	***P***
***VERB***	1,21	22.45	< 0.0005	3.69	n.s.
***SUBJ***	1,21	1.53	n.s.	7.17	< 0.05
***VERB x SUBJ***	1,21	4.47	< 0.05	29.28	< 0.0001
***VERB x REG***	2,42	26.46	< 0.0001	19.31	< 0.0005
***VERB x SUBJ x REG x HEM***	2,42	0.60	n.s.	5.77	< 0.05

Note: The above Global ANOVA was carried out with the first factor VERB (*–s* suffix on the verb, No–*s* suffix on the verb), the second factor SUBJ (Singular subject NP, Plural subject NP), the third factor REG (Anterior, Central, Posterior), and the fourth factor HEM (Left, Right).

### Mathematical reasoning experiment

In addition to the auditory ERP experiment described above, available study participants also took part in a mathematical reasoning experiment aimed at determining whether the effect of verbal–*s* identified in the ERP portion of the study impacts mathematical reasoning. Stimuli for this second experiment were designed to mimic the familiar mathematical “word problems” given in elementary school albeit in a linguistically controlled manner.

### Methods

#### Participants

Eighteen of the twenty-two study participants took part in the experiment. Participant selection was based solely on prior participation in the ERP experiment and availability. No other selection criteria were used.

#### Stimuli

As noted above, the stimuli for the mathematical reasoning experiment were constructed so as to resemble the “word problems” commonly given to elementary school students. Such problems are given both orally and in written form on classroom tests, homework assignments, and as a part of psychoeducational batteries like the Woodcock Johnson [26]. Presented orally, the stimuli for the current experiment comprised 60 pre-recorded mathematical word problems, examples of which are provided in Tables 9 and 10. Following a 2 x 3 factorial design, the stimuli varied in 1) Problem Type, whether the problem was an addition or subtraction problem (Tables 9 and 10, respectively), and 2) Sentence Type, the grammar of sentences used to pose the problem. Consistent with SCE, but not AAE grammar, a third of the questions– 20 problems total, 10 addition, 10 subtraction–used sentences that paired singular subject NPs with–*s* suffixed verbs (MSS in Tables 9 and 10). Consistent with AAE, but not SCE grammar, a third of the questions–again, 10 addition and 10 subtraction–used sentences that paired singular subject NPs with suffix-less verbs (MSN in Tables 9 and 10). Finally, consistent with both SCE and AAE grammar, the remaining 10 addition and 10 subtraction questions used sentences that paired plural subject NPs with suffix-less verbs (MPN in Tables 9 and 10). To distinguish them from those in the auditory ERP experiment, the condition labels used here (MSS, MSN and MPN) all begin with “M” for math. Like the ERP labels, however, the following “P” or “S” stands for plural or singular subject, and the trailing “S” or “N” stands for–*s* suffix on verb or no suffix. In addition to the linguistic controls just described, the difficulty level of the math questions was controlled so that they would match that of problems the participants–all 2nd graders–would typically encounter in school. Specifically, solving each problem required either the addition or subtraction of two single digit numbers each ranging from 1 to 9, with the resulting sum or difference also being a single digit number that ranged from 1 to 9 (see Tables 9 and 10 for examples).

**Table 9:** Example auditory stimuli for the mathematical reasoning experiment (addition Problems).

Condition	Label	Addition Problem
**Singular Subject NP,–*s* on Verb**	**MSS**	At the restaurant, the cook makes chili. In it, he uses two green peppers and he uses two red peppers. How many peppers does he use in all? [2+2 = 4]
**Singular Subject NP, No–*s* on Verb**	**MSN**	At the restaurant, the cook make chili. In it, he use two green peppers and he use two red peppers. How many peppers do he use in all? [2+2 = 4]
**Plural Subject NP, No–*s* on Verb**	**MPN**	At the restaurant, the cooks make chili. In it, they use two green peppers and they use two red peppers. How many peppers do they use in all? [2+2 = 4]

Note: The correct calculation for each example is indicated in square brackets after each question.

**Table 10:** Example auditory stimuli for the mathematical reasoning experiment (subtraction Problems).

Condition	Label	Subtraction Problem
**Singular Subject NP,–*s* on Verb**	**MSS**	At the restaurant, the cook makes chili. In it, he uses three peppers. One of the peppers he uses is green and the rest are red. How many red peppers does he use? [3–1 = 2]
**Singular Subject NP, No–*s* on Verb**	**MSN**	At the restaurant, the cook make chili. In it, he use three peppers. One of the peppers he use is green and the rest are red. How many red peppers do he use? [3–1 = 2]
**Plural Subject NP, No–*s* on Verb**	**MPN**	At the restaurant, the cooks make chili. In it, they use three peppers. One of the peppers they use is green and the rest are red. How many red peppers do they use? [3–1 = 2]

Note: The correct calculation for each example is indicated in square brackets after each question.

The methods used to record the questions were the same as those used in the previously described auditory ERP experiment. A female speaker, different from the speaker that recorded the target ERP stimuli, spoke the math question stimuli. Across all conditions, both pitch and duration of the questions were matched as much as possible. Due to necessary differences in their wording, the subtraction problems were significantly longer than the addition problems (*F*(1,9) = 75.58, *P* < 0.0001) (see Table 11). No other difference was found between the different conditions in Sentence Type, and there was no significant interaction between Sentence Type and Problem Type (*F*’s < 1.80).

**Table 11:** Mean durations of the auditory stimuli for the mathematical reasoning experiment (seconds) with their SDs (in parentheses).

		Mean Duration (ms)	
Condition	Label	Addition Problem	Subtraction Problem
**Singular Subject NP,–*s* on Verb**	**MSS**	15.28 (1.81)	17.86 (1.95)
**Singular Subject NP, No–*s* on Verb**	**MSN**	15.15 (1.80)	17.93 (2.00)
**Plural Subject NP, No–*s* on Verb**	**MPN**	15.33 (1.66)	17.74 (2.03)

#### Procedures

Sessions for the ERP and mathematical reasoning experiments took place on separate days in the same quiet room at the participants’ elementary school. The order of experiments was kept constant for all participants: ERP session first, followed by the mathematical reasoning session. Ahead of the mathematical reasoning sessions, each participant was randomly assigned to one of four counterbalanced lists of stimuli. Each list was divided into two blocks, a block of addition problems and a block of subtraction problems. Half of the participants answered the addition problems first, and the subtraction problems second. The other half answered the subtraction problems first followed by the addition problems. In each session, after ensuring that the participant was ready to begin, the experimenter played pre-recorded versions of the math questions to be answered. After hearing a question, the participant spoke his or her answer, and the experimenter recorded it on a scoring sheet. Participants were permitted to listen to the same question up to three times. If a participant could not arrive at an answer to a question, he or she could respond “I don’t know” and the experimenter would move on to the next question. No feedback was provided to participants after answering questions.

The mathematical reasoning data were analyzed as follows. For each participant, percent correct response measures were calculated for each problem type for each sentence type. Unanswered questions and those answered with “I don’t know” were included in the data analysis as false responses. Note that response times were not recorded in the mathematical reasoning experiment and therefore not included in the analysis. To examine the effects of problem type, whether the question a participant was asked required addition or subtraction to solve; and sentence type, whether the problem was posed in a manner consistent with SCE (but not AAE) grammar, AAE (but not SCE) grammar, or both grammars; repeated measures 2 x 3 ANOVAs with the first factor PROBLEM TYPE (addition, subtraction) and the second factor SENTENCE TYPE (MSS, MSN, MPN) were carried out using error terms based on participant (*F*_*1*_) and item variability (*F*_2_). In addition to these statistical tests, based on our predictions, planned contrast tests were conducted to compare the percent correct response for MSS, the sentence type ungrammatical in AAE, to the percent correct response for the mean of MSN and MPN, the sentence types grammatical in AAE.

## Results

Table 12 presents the results of the mathematical reasoning experiment. Repeated measures 2 x 3 ANOVAs showed a significant effect of PROBLEM TYPE for both the participant and item analyses. Questions that required solving addition problems to answer were, on average, answered more accurately than those that required solving subtraction problems (Addition vs. Subtraction: 85.18 vs. 65.37%: *F*_*1*_(1,17) = 21.93, *P* < 0.0005, *F*_*2*_(1,9) = 29.26, *P* < 0.0005). More importantly, the above ANOVAs indicated that an effect of SENTENCE TYPE was significant for both the participant and item analyses (*F*_*1*_(2,34) = 4.73, *P* < 0.05, *F*_*2*_(2,18) = 4.09, *P* < 0.05). The significant effect of SENTENCE TYPE likely stemmed from the fact that questions written using MSN and MPN sentences, the two sentence types consistent with AAE grammar, were answered more accurately than those written using MSS sentences, the sentence type inconsistent with AAE grammar (77.50% on average vs. 70.83%). To test this conjecture, a planned contrast test was performed. The test supported the general data pattern that questions that used grammatical sentences were answered more accurately than those that used ungrammatical sentences by showing that the mean of the MSN and MPN measures was significantly greater than MSS for the participant analysis (*F*_*1*_(1,17) = 6.88, *P* < 0.05, *F*_*2*_(1,9) = 4.80, *P* = 0.06). No effect other than those reported above reached significance.

**Table 12:** Mean percent correct response (%) with SDs (in parentheses) for the math experiment.

		Correct Response (%)	
Condition	Label	Addition Problem	Subtraction Problem
**Singular Subject NP,–*s* on Verb**	**MSS**	81.66 (17.90)	60.00 (25.89)
**Singular Subject NP, No–*s* on Verb**	**MSN**	87.77 (15.55)	68.33 (23.82)
**Plural Subject NP, No–*s* on Verb**	**MPN**	86.11 (14.60)	67.77 (23.65)

## Discussion

Together, the results of the two experiments described in this paper argue that SCE verbal–*s* poses sentence processing problems for AAE speaking 2nd graders–problems that, in turn, can undermine these students’ performance on orally administered mathematical reasoning tasks. To that point, consistent with previous research, the children in the current study were less accurate answering addition and subtraction word problems that used verbal–*s* than those that did not, suggesting that trying to process what we assumed for them was a less than fully available and ungrammatical morpheme hindered their performance on the task (cf. [9, 10]). And, designed to investigate the cognitive effects of verbal–*s* more directly, the study’s auditory ERP experiment likewise provides considerable support for our contention that verbal–*s* was indeed less than fully available to these children and lies outside of AAE grammar. Compared to AAE grammatical sentences that paired a singular NP subject with a suffix-less verb (SN), hearing sentences that paired singular NP subjects and–*s* marked verbs (SS) led the children to produce a bilateral anterior-central negativity in the 200–400 ms time window that was established when the ERPs were time-locked to the onset of the target position (see Table 1 for the experimental conditions and Fig 4 for the experimental results). Following this early negativity, these same sentences (SS), again, compared to their clearly grammatical counterparts (SN), elicited a long lasting bilateral anterior-central negativity in the 400–900 ms time window. As discussed further in the sections to follow, the two negativities suggest the children identified verbal–*s* in these sentences as ungrammatical, and that it increased their processing load due to its lack of place in a tense and agreement system accessible to them.

Distinct from this type of sentence, sentences formed by combining a plural (as opposed to singular) subject NP with an–*s* marked verb (PS), themselves ungrammatical in AAE, elicited a different response. Compared to their closest grammatical counterparts–sentences formed from plural subject NPs with suffix-less verbs (PN)–these sentences elicited a bilateral early anterior-central negativity (200–400 ms) followed by a bilateral late central-posterior positivity (400–900 ms). We interpret these two different patterns–the early negativity followed by a long lasting negativity, and the early negativity followed by a late positivity–as reflecting two different strategies available to AAE speaking children who encounter verbal–*s*, the former employed when they are unable to identify verbal–*s* as having a place in their tense and aspect system at all, the latter when it is misidentified as a plural.

In what follows, we discuss the ERP signatures outlined above in greater detail before addressing the math study results.

### The ERP effects

#### The bilateral early anterior-central negativities

We initially predicted that due to its limited cognitive accessibility and ungrammaticality in their home dialect, AAE speaking children would identify the verbal–*s* in both singular subject sentences like *Mary drinks coffee* (SS) and plural subject sentences like *The workers drinks coffee* (PS) as an error. In both cases, we assumed that compared to their closest error-free counterparts, error detection would be reflected in the children’s producing a relatively early anterior negativity (SS vs SN and PS vs. PN). However, we also expected the two negativities to differ from one another, and in the case of the singular subject sentences (SS) to differ from the LAN most commonly associated with morphosyntactic error detection (e.g., [55]). This electrophysiological signature, normally part of a bi-phasic pattern of LAN followed by P600, is associated with the misuse of familiar morphology that results, for example, from morphological agreement errors (for an overview, see e.g., [32]). In contrast, the verbal–*s* that AAE speaking children encounter in a sentence like *Mary drinks coffee* is unfamiliar in the sense that it is less cognitively accessible to them. As previously noted, we believe this type of error is more akin to the kind of structure building error associated with a phrase structure violation than an agreement error. Unable to give it a semantic representation, AAE speaking children presented with verbal–*s* in this context are, in effect, given a building block of morphosyntactic structure without a blueprint for its use. A sentence like *The workers drinks coffee*, on the other hand, differs in that the plural–*s* on the subject, *the workers* in this case, makes that fully accessible morpheme and its rules of use salient. We hypothesized that hearing sentences of this type, the young AAE speakers in our study would be “tricked” into thinking that verbal–*s* was a misplaced plural. Thus, it would be detected as a morphological agreement error rather than a morphosyntactic structure building error, a distinction that should be visible in the ERP signatures.

The above expectations were largely met. Although neither type of ungrammatical sentence (SS or PS) elicited an ELAN, both did elicit a bilateral early anterior-central negativity. The broader than expected distributions–bilateral rather than left-lateralized, and anterior-central rather than anterior–are likely due to the young ages of our study participants. Compared to adults, children often need to use a greater proportion of their cognitive resources to perform the same linguistic tasks (e.g., for an overview, see [79, 80]). Our ERP predictions were based on adult processing studies; all our participants, however, were 2nd graders.

Although the verbal–*s* in both sentence types under discussion triggered a bilateral early anterior-central negativity, the two negativities were predicted and found to be different from one another. Statistical analysis showed that the amplitude of the negativity triggered by the verbal–*s* in singular subject sentences like Mary drinks coffee (SS) was higher than that that triggered by the–*s* in plural subject sentences like *The workers drinks coffee* (PS) (see the results of all condition comparisons in S2 Fig). We interpret this difference in ERP amplitude as reflecting different morphosyntactic processes, the former the detection of a morphosyntactic structure building error, the latter the detection of a misplaced morpheme.

At 200 ms, the bilateral anterior-central negativity we associate with the detection of misplaced/misused morphology (PS) is slightly earlier than the LAN we equate it with in the literature (e.g., [43, 44, 51–59]). The earlier onset time is most likely due to a difference in where the ERPs were time-locked: in the current ERP experiment, the ERPs were time-locked directly to the critical morpheme onset (verbal–*s*), whereas they were time-locked to the critical word onset in the previous studies.

#### The late ERP effects

Further evidence that the bilateral anterior-central negativity that verbal–*s* induced in plural subject sentences (the PS condition) reflects the detection of an agreement-like morphological error comes from the late time window ERP effects. Compared to their grammatical counterparts (PN), sentences of this type elicited a bilateral late central-posterior positivity in the 400–900 ms time window. We identify this late positivity with the P600 effect often found in adult studies of morphological error. Following a LAN, the P600 effect has been taken to reflect either the repair of a misidentified morpheme or the engagement of a general integration process associated with the repair of some element within in an ungrammatical sentence.

In contrast, the verbal–*s* in singular subject sentences (the SS condition) did not elicit a P600. In line with our predictions, the bilateral early anterior-negativity (200–400 ms) in these sentences was followed by a longer lasting bilateral anterior-central negativity in the 400–900 ms time window. We view this second negativity as an indicator of the increased working memory load the children incurred as a result of not having a full representation for verbal–*s* in their morphological inventories and therefore not having a clear procedure for coping with its presence. Long lasting (or sustained) negativities have been found in previous studies that tested both the online processing of sentences (e.g., [71, 89–92] and other experimental tasks that required increased working memory loads (for an overview, see e.g., [93]). Both the early and late time window effects, then, suggest that AAE speaking children can approach verbal–*s* in two ways: they can misidentify it as a plural marker (the case of PS) or they can fail to identify it as a part of a system they control at all (the case of SS).

One might imagine a different explanation for the ERP pattern we observed in the PS condition. Instead of misidentifying verbal–*s* as a plural marker, the same or a similar pattern might be thought to arise due to the children’s misidentifying the plural–*s* on the subject noun as a possessive (e.g., *the worker’s* for *the workers*), only to have this initial hypothesis disconfirmed by the verb phrase. As is the case with verbal–*s*, it has been argued that possessive–*s* (in the attributive position) is not a part of AAE grammar. Sentences like *This is John cup* (in SCE, *This is John’s cup*) are grammatical and common in both adult and child AAE [4, 25]. However, unlike with verbal–*s*, comprehension studies suggest that, in the main, young AAE speaking children give this–*s* an SCE interpretation [94]. Although unlikely, it is possible, then, that AAE children’s first impression of the plural would be that it is a properly placed SCE possessive, and with that morphology not fully a part of their grammar, they would produce a LAN-like effect. A P600 would, then, follow the verbal–*s*, as the verb phrase makes it clear that the initial–*s* is not a possessive and, therefore, must be reanalyzed reanalysis must occur. This account, however, will not explain the results of the current experiment, as in the sentences presented to the children, the subject noun phrase was always separated from the verb phase by a prepositional phrase (e.g., *from Durham* in the sentence *Six kids from Durham eats chocolate cake*), the prepositional phrase making clear that the initial–*s* is not a possessive well before the verb phrase containing verbal–*s* is introduced. Misidentification of verbal–*s* as the plural remains the most plausible account of the ERP effects that we observed.

### The math study

The ERP results and the analysis we have given them above invite the questions *How did the children in the study interpret verbal–s in the mathematical word problems they heard*? and *What processing strategy or strategies did they use to cope with it*? In our view, it is unlikely that, in the main, the participants in this study mistook the verbal–*s* in the math problems they were presented for the plural. Data from the ERP experiment suggest that the same children were only led to interpret verbal–*s* as a plural when presented sentences that are ungrammatical in both AAE and SCE, a kind of sentence they would not have heard in school and did not hear in the mathematical reasoning experiment. Although the data point away from the children’s having mistaken verbal–*s* for the plural and towards a response to the morpheme’s ungrammaticality as the source of its effect on solving math problems, there is still no direct evidence that this is the case. The ERP and math portions of the current study were conducted as separate experiments. Without further work, it remains a possibility that the children did mistake verbal–*s* for the plural–*s* especially as it has been suggested in the literature that young AAE speakers can in fact mistake verbal–*s* for the plural marker in sentences that are grammatical in SCE [2, 8, 94].

SCE verbal–*s* marks both number and tense. In terms of number, it is licensed by third person singular subjects (*I agree*, *You agree*, *He/She/It agrees*). With regard to tense, it only occurs in the present (*He agrees*, *He agreed*, *He will agree*). As part of a series of experiments examining AAE speaking children’s understanding of SCE verb endings, Torrey (1983) [94] used separate picture meaning tasks to test AAE speaking 2nd graders’ knowledge of these grammatical contrasts, and in doing so test their knowledge of the meaning of the verbal–*s* morpheme itself. In her test of the past/present distinction, participants would, for example, hear either the sentence *The man hits the dog* or *The man hit the dog*, and then be asked to choose which of two pictures best represented the sentence they had just heard, a picture of a man in the act of hitting a dog with a stick, or one of a man waving a stick while a dog was running away. To test the singular/plural distinction, participants were read either a sentence like *The cat splashes* or one like *The cats splash*, the former pairing a singular subject with an–*s* suffixed verb, the latter pairing a plural–*s* marked subject with a suffix-less verb. Key to the experimental design, the verb that followed the subject noun always began with the same voiceless alveolar sibilant sound as the plural–*s*, thus, acoustically masking the plural, and leaving the absence or presence of verbal–*s* as the only indicator of number. Neither Torrey, whose data were collected from AAE speakers in Harlem NY, nor Ball (1994) [8], who replicated Torrey’s (1983) [94] study in a Detroit MI area school, found their participants could reliably infer number from the SCE morpheme. Torrey reports a 5% rate of doing so and Ball a 60.3% rate in one group of speakers, and 64.3% rate in another. These numbers, especially the very low numbers from the Torrey study, lead Labov and Baker (2015) [2] to conclude that in these experiments, AAE speaking children more often than not inferred a plural meaning from SCE verbal–*s*.

Following this line of thought, they interpret the results of J. M Terry et al. (2010) [9] differently than do its authors. As noted in the introduction to the current paper, that work used a Bayesian Markov Chain Monte Carlo Method (MCMC) to estimate, among other things, the effect of verbal–*s* on a group of AAE speaking 2nd graders’ performance on the WJ-R test of Applied Problems. Its chief finding was that the more instances of verbal–*s* there were in a test question, the less likely children were to answer it correctly. Largely motivating the explanation given for the data in the current set of experiments, the study’s authors argued that this effect was the result of the children’s having to bear an increased working memory load that robbed them of cognitive resources that would otherwise have been available for problem solving, the load itself resulting from the children’s failure to fully recognize the verbal–*s* morpheme. Labov and Baker suggest a different cause: that the children systematically misidentified verbal–*s* as the plural marker, and rather than discarding its plural meaning for having been inappropriately placed on the verb, used it to give a plural interpretation to the subjects of the sentences in which it occurred. At first blush, the Labov-Baker hypothesis would seem to have the advantage of straightforwardly explaining the surprisingly high cost many of the children seem to have paid for encountering verbal–*s*. The MCMC model used in the study predicted that for roughly 15% of the children tested, the average child would answer 9% more questions correctly if it were not for the effect of verbal–*s*. Mistaking verbal–*s* for the plural is a processing misstep that potentially could have severe consequences for scores on mathematical reasoning tests. Although some of the WJ-R applied math problems involve telling time or other number related activities that do not necessarily require performing calculations, the majority are calculation dependent. Successful completion of any calculation dependent problem requires accurate counts of the numbers involved in its calculations, and interpreting singularity where plurality is called for–to the extent that it misrepresents those counts–would certainly lead to incorrect answers and lower scores.

This does not seem to be what happened in this case, however. Of the problems on form A of the WJ-R, the form that was used in the experiment in question, only two (problems 31 and 36) make use of verbal–*s* and, at the same time, are written so that subject number is arguably critical to the calculation. Subject number plays no role whatsoever in the calculations required to solve the vast majority of the WJ-R problems. That said, the Applied Problems section of the WJ-R is “ceiling tested”, that is, each student is asked questions until that student incorrectly answers a specified number of them in a row and the test is stopped. So, although presumably small, it is difficult to estimate the effect of these two problems on the students’ performance as a whole. Not every student was necessarily asked these two questions. Data collected as a part of the current study present a clearer picture, and a more compelling argument against Labov and Baker’s hypothesis. In the current study, none of the math questions given to participants required calculations that used counts of subjects. The numerical information required for all calculations was contained in either object or adjunct positions within the problems’ sentences. The addition problem below is an example.

On Fridays, the boy next door stacks books. All by himself, he stacks four books on the top shelf and he stacks three books on bottom shelf. How many books does he stack in all?

Whether there is one boy or many, the calculation above remains the same. The total number of books stacked is seven. All of the questions asked in this experiment were similar in this respect. The negative performance effects of verbal–*s* in it, then, cannot be explained by the children’s having systematically confused verbal–*s* for plural–*s* in such a way as to directly interfere with the counts that fed into their calculations. Thus, this style of explanation not only fails to add any explanatory power when it comes to the magnitude of the effect, but it leaves the effect itself to be explained either by the surprise that comes from finding an–*s* affixed to the end of a verb or the processing required to cope with it. “Coping with it” might take the form of attaching a plural meaning to the subject noun or discarding the morpheme and its meaning from the sentence processing altogether.

Although a question not answered here, whether or not the AAE speaking children in the current study mistook verbal–*s* for the plural in math problems they answered has important practical implications. At stake is the generality of the over-all problem verbal–*s* presents. An explanation of the effect of verbal–*s* found in the current study based on children’s confusing verbal–*s* and plural–*s*, leads one to expect that effects like these will be rare. It is an unhappy accident that verbal–*s*, a marker of the singular, shares its phonological form with the plural marker in both SCE and AAE. This particular kind of morphological mismatch is unlikely to occur elsewhere between the two dialects or between other dialects. If, on the other hand, the verbal–*s* effect is driven primarily by the morpheme lying outside of children’s grammar, and not by its homophony with some other morpheme with a critically important meaning, expectations change. Should this analysis turn out to be correct, one would expect a much greater number of similar mismatch problems in and across dialects and a more general strategy of intervention would be warranted.

## Conclusions

The experiments described herein were designed with a very practical goal in mind: to better our understanding of which differences between AAE speaking children’s home and school dialects have a significant impact on their educational achievement and why. We believe that the complexity of this issue demands that in addition to research that aims to correlate general measures of AAE use with academic achievement, examination of specific mismatches between AAE and SCE on specific tasks is required if that goal is to be met. Our finding that SCE verbal–*s* depresses AAE speaking 2nd graders’ scores on orally administered math tests argues that non-reading specific mechanisms are at play in at least some dialectal difference effects on measures of academic achievement, and further suggests that they may even play a role in reading difficulties. The neurocognitive findings we report argue that the effect of verbal–*s* on math tests results from an increase in working memory load that itself stems either from a failure on the part of AAE speaking children to fully recognize the morpheme or from their trying to process it after mistaking it for a plural marker. Together these findings open new space for generating hypotheses about role of dialect in education.

## Supporting information

S1 Fig Results of 4 x 3 x 2 Global ANOVA for the two selected time windows (200–400 ms and 400–900 ms) with interaction resolutions. (TIF)

S2 Fig Results of condition comparisons for the two selected time windows (200–400 ms and 400–900 ms) with interaction resolutions. (TIF)

S3 Fig Results of 2 x 2 x 3 x 2 Global ANOVA for the two selected time windows (200–400 ms and 400–900 ms) with interaction resolutions. (TIF)
