# Supplementary figures and images for: African American English speaking 2nd graders, verbal–s, and educational achievement: Event related potential and math study findings
Source: PLoS One. 2022 Oct 20;17(10):e0273926. doi: 10.1371/journal.pone.0273926 (PMC9584506; doi:10.1371/journal.pone.0273926)

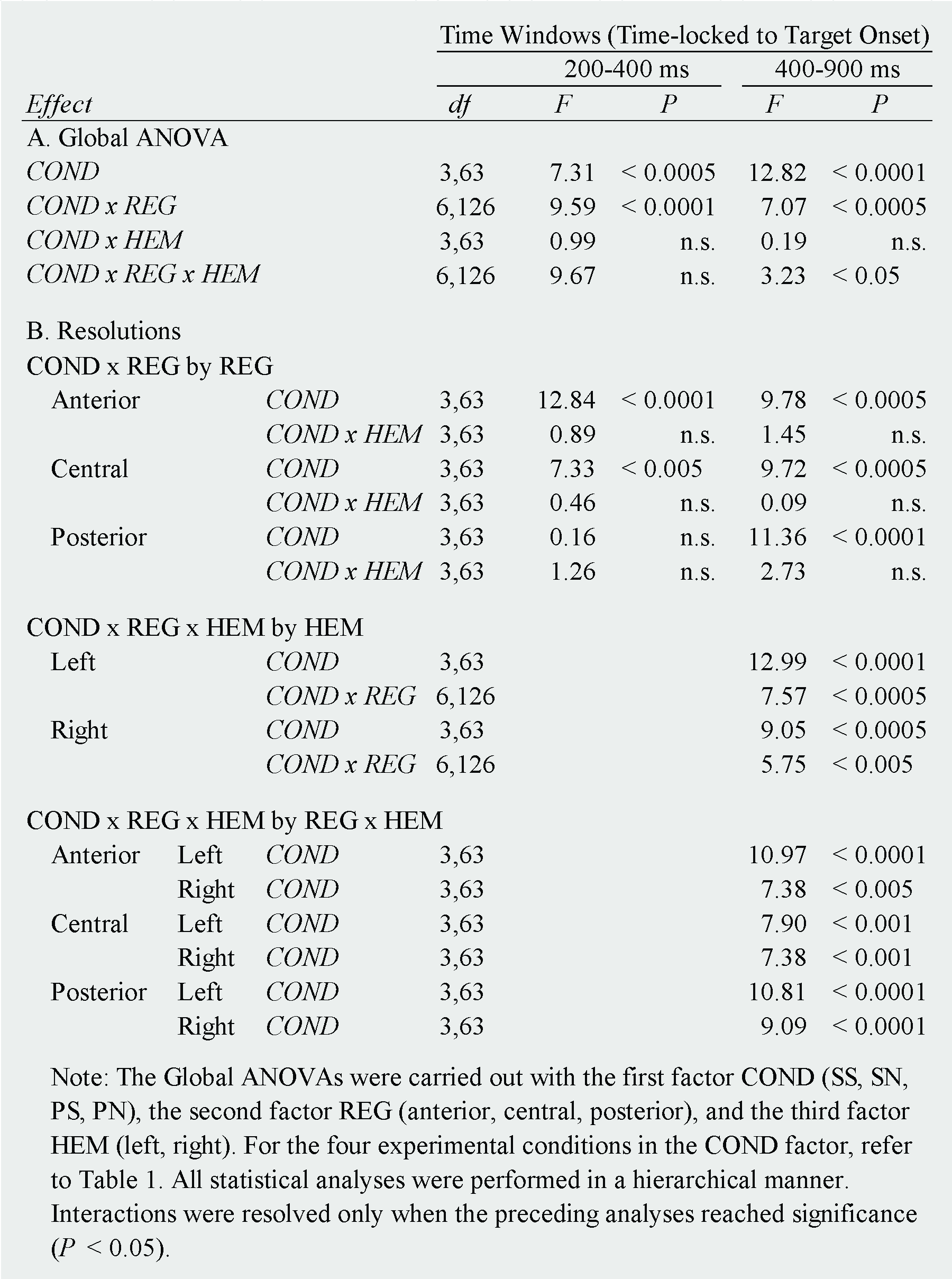

Supplement: S1 Fig — (TIF) [file pone.0273926.s001.tif]

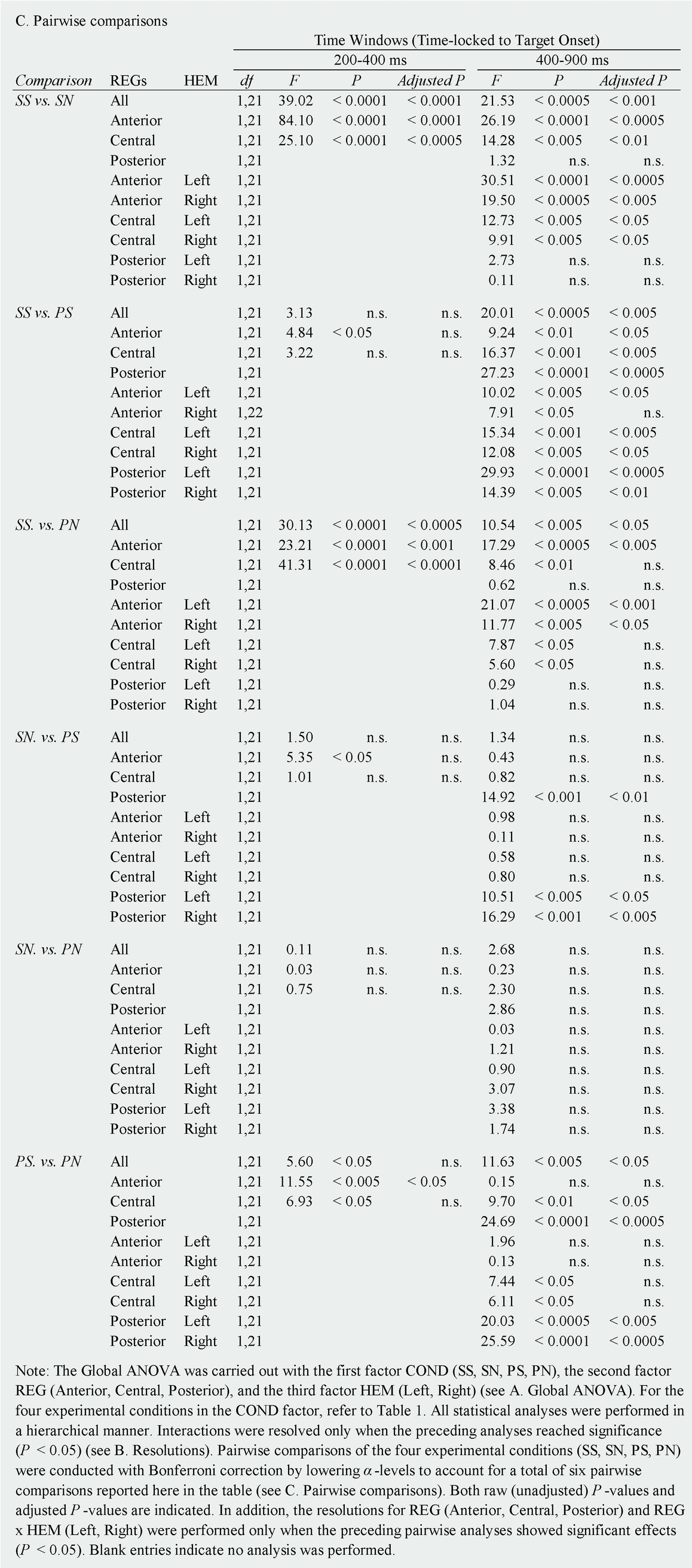

Supplement: S2 Fig — (TIF) [file pone.0273926.s002.tif]

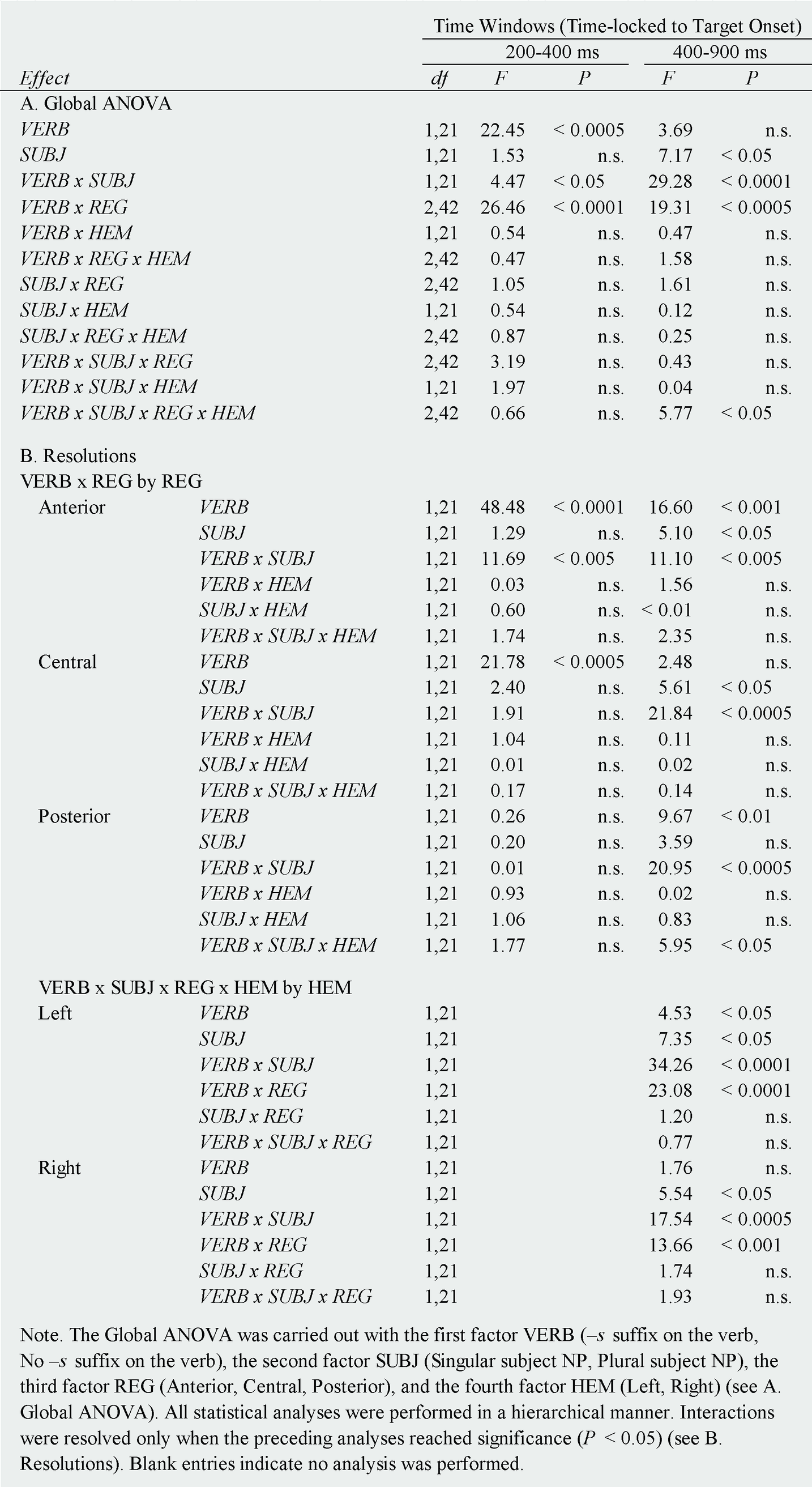

Supplement: S3 Fig — (TIF) [file pone.0273926.s003.tif]
